# Integrating single-cell transcriptomics into model systems to decode neurodegeneration in amyotrophic lateral sclerosis

**DOI:** 10.3389/fncel.2026.1911064

**Published:** 2026-10-26

**Authors:** David Orsulik, Teresa Niccoli

**Affiliations:** 1 Department of Genetics, Evolution and Environment, Institute of Healthy Ageing, University College London, London, United Kingdom; 2 UK Dementia Research Institute, University College London, London, United Kingdom

**Keywords:** ALS, C9, *Drosophila*, model systems, repeat expansion, single cell RNA-sequencing, TDP43

## Abstract

Amyotrophic lateral sclerosis (ALS) is a fatal neurodegenerative disease characterised by motor neuron degeneration. Several genetic risk factors, including mutations in *C9orf72*, *SOD1*, *TARDBP* and *FUS* have been linked to disease. However, the precise cellular pathogenic mechanisms of ALS remain poorly understood, impeding the development of effective therapeutics. ALS model systems, ranging from *Drosophila*, mice to human induced pluripotent stem cells (hiPSCs), are powerful research platforms for decoding these mechanisms, with their high genetic tractability allowing for the tracking of disease across the life course. Recently, the application of single cell resolution approaches have allowed the dissection of the heterogenous nature of cellular response to disease in these models, revealing novel ALS pathogenic mechanisms and therapeutic targets. In this Review, we explore the unique benefits and challenges of using single-cell RNA-sequencing (scRNA-seq)/single-nucleus RNA-sequencing (snRNA-seq) analysis of *in vivo* and *in vitro* ALS model systems to identify disease pathways and drive our mechanistic understanding of ALS forward. By using single cell approaches in models of ALS, researchers can identify novel, druggable, cellular pathways acting across all disease stages that may be obscured in late-stage post-mortem tissue.

## 1 Introduction

### 1.1 The genetics and pathophysiology of ALS neurodegeneration

Amyotrophic lateral sclerosis (ALS) is a devastating neurodegenerative disease, for which there is currently no effective cure and whose mechanisms remain poorly understood (Mizielinska et al., 2025). Most ALS cases (90%) are sporadic (sALS), but approximately 10% of cases are familial (fALS) (Bradford and Rodgers, 2024). Several genetic risk factors are linked to ALS development, with mutations within *C9orf72, SOD1, TARDBP* and *FUS* being the best studied (Table 1) (Bradford and Rodgers, 2024).

**Table 1:** Summary of the ALS-linked mutations in *C9orf72*, *SOD1*, *TARDBP* and *FUS* and associated model systems.

Genetic risk factor	Pathogenic mutation	Model No.	Associated model systems	Reference
*C9orf72*	GGGGCC hexanucleotide repeat expansion (>30 repeats) (Balendra and Isaacs, 2018; Sellier et al., 2024)	1	*Drosophila* (C9-DPR)	Mizielinska et al. (2014)
		2	Mouse (C9-BACexp)	O'Rourke et al. (2015)
		3	hiPSC (C9-ALS patient derived)	Ho et al. (2021)
*SOD1*	Missense point mutations: (A5T, A5V, L9V, G11V, D12Y, F21C, L39V, H49Q, D77Y, G86R, D91A, G93A, G94R, G94C, G94S, E100G, I114T, R116G, T138R, L145F, L145S, G148D, I150T) Benatar et al. (2025)	4	*Drosophila*	Şahin et al. (2017)
		5	Mouse	Gurney et al. (1994)
		6	hiPSC	Namboori et al. (2021)
*TARDBP*	Missense point mutations: (D169G, N267S, G287S, G290A, G294A, G294V, G295S, G295R, G298S, M311V, A315T, Q331K, S332N, G335D, M337V, Q343R, N345K, G348C, N352S, R361S, P363A, Y374X, S379P, S379C, A382T, A382P, I383V, N390S, N390D, S393L) Lagier-Tourenne and Cleveland (2009)	7	*Drosophila*	Langellotti et al. (2016)
		8	Mouse	Swarup et al. (2011)
		9	hiPSC	Schweingruber et al. (2025)
*FUS*	Missense point mutations (Q140R, Q156E, G191S, R216C, R234L, R244C, R495X, R514G, R514S, G515C, H517Q, R518K, R521H, R521G, R521C, R522G, R524S, R524T, P525L) Xiao et al. (2024)	10	*Drosophila*	Chen et al. (2011)
		11	Mouse	Qiu et al. (2014)
		12	hiPSC	Schweingruber et al. (2025)

The list of ALS-associated mutations and model systems shown here directly relate to the studies discussed in this Review and is not exhaustive. For a more comprehensive list of *Drosophila* ALS model systems see https://flybase.org (Jenkins et al., 2022). For a more comprehensive list of mouse ALS models see https://www.alzforum.org/research-models/als. For a more comprehensive list of human-derived induced pluripotent stem cell (hiPSC) ALS models see (Dawoody Nejad and Pioro, 2025). For a more comprehensive list of ALS genes see https://alsod.ac.uk/ (Abel et al., 2012).

These genetic risk factors are thought to drive several pathological hallmarks of ALS including polyubiquitinated pathological inclusions, containing a number of proteins, most commonly TDP43 (Blokhuis et al., 2013; Jankovska and Matej, 2021). These can lead to cellular homeostasis loss, ultimately resulting in motor neuron (MN) degeneration and structural network integrity loss across the motor cortex (Nihei et al., 1993; Verstraete et al., 2010). In this Review we introduce ALS pathophysiological mechanisms, along with common ALS animal and cell models, subsequently showcasing how single cell RNA-sequencing (scRNA-seq) and single-nucleus RNA-sequencing (snRNA-seq) techniques, in model systems in particular, are being used to improve our understanding of ALS neurodegeneration mechanisms and help identify potential therapeutic avenues. Please note that the studies discussed here and in Table 2 were selected as a representative list of the field at time of writing and are not exhaustive. The studies discussed were selected to provide adequate coverage across both *in vivo* (mouse and *Drosophila*) and *in vitro* (cell-based) ALS model systems, giving the reader a broad view of how scRNA-seq has been applied across multiple models.

**Table 2:** Summary of ALS-focused scRNA-seq studies in model systems covered in this review.

Model system	ALS mutation	Platform	Isolation strategies	Research type	Main biological finding	Reference
hiPSC	*C9orf72*-HRE	ddSEQ	Microfluidic	Primary	hiPSC-MN sampling during *in vitro* differentiation helped identify early and predictive ALS MN expression. These include dysregulated processes linked to differentiation, cell-type specification and homeostatic activity, highlighting them as potential predictive markers of disease-associated MNs.	Ho et al. (2021)
hiPSC	SOD1-E100G	Fluidigm C1	Microfluidic	Primary	Transcriptional regulatory network analyses implicated several developmental pathways in ALS MN gene dysregulation. Examples include increased TGF-β signalling, with downstream SMAD2 overactivation driving neurodegeneration.	Namboori et al. (2021)
hiPSC	*TARDBP*-M337V, FUS-P525L, FUS-R495X	Smart-seq2	FACS	Primary	Single-cell analyses highlighted mitochondrial dysregulation as a common early signature across both *TARDBP* and *FUS*-ALS, with broad downregulation of ATP synthesis-coupled electron transport and oxidative phosphorylation prior to both TDP43 and FUS cytoplasmic mis-localization.	Schweingruber et al. (2025)
hiPSC	*C9orf72*-HRE	10X Genomics	FACS	Primary	snRNA-seq analyses of patient-derived microglial xenografts revealed *C9orf72*-HRE and knockout impairs microglial immune responsiveness. These microglia downregulate immunoreactive HLA genes whilst upregulating homeostatic genes, preventing their transition to a reactive state.	Masrori et al. (2025)
hiPSC	*C9orf72*-HRE	10X Genomics	Microfluidic	Primary	scRNA-seq of LPS-stimulated microglia/MN co-cultures further suggest *C9orf72*-HRE reduce microglial reactivity. Findings revealed reduced cytokine activity as well as a complete loss of a LPS-responsive microglial subpopulation.	Gao et al. (2026)
Mouse	SOD1-G93A	MARS-seq	FACS	Primary	scRNA-seq on spinal cord tissue during early and late disease stages identified a previously unknown subpopulation of DAMs from a heterogeneous cellular pool. A 24% increase in DAM proportions in late-stage mice further suggests these cells accumulate during disease progression.	Keren-Shaul et al. (2017)
Mouse	SOD1-G93A	Drop-seq	Microdroplets	Primary	ScRNA-seq on mouse pontine brainstem identified sodium channel gene upregulation and potassium channel gene downregulation in MNs. Together, these channel dysregulations may drive the hyperexcitability and calcium dysregulation phenotypes that likely contribute to MN death.	Liu et al. (2020)
Mouse	SOD1-G93A	Various	N/A (Computational integration with human data)	Secondary	EWCE comparisons across mouse and human scRNA-seq datasets helped identify novel spinal cord α- and γ-MN subpopulations involved in human ALS pathogenesis. Their findings indicate γ-MNs associate primarily with genes known to carry ALS causal mutations like *SOD1* and *VCP*, whilst α-MNs associate with susceptibility genes.	Liu et al. (2023)
Mouse	*C9orf72*-HRE, *C9orf72*-knockout	10X Genomics	Single nuclei isolation via ultracentrifugation	Primary	C9-BAC and *C9orf72* knockout mouse frontal cortex analyses suggest progressive C9orf72 loss results in the downregulation of ribosomal proteins and biogenesis machinery, leading to a cellular stress response linked to proteotoxicity.	Ma et al. (2023)
Mouse	SOD1-G93A	Various	N/A (Computational integration with human data)	Secondary	Cross-comparisons between human and mouse data identified several novel potential ALS biomarkers for non-neuronal microglia, including CALM1, CALM2 and HSPA1A. These analyses also highlighted that microglia dysregulate pathways related to energy metabolism, with this correlating with reduced astrocytes interaction and worsening neuroimmune dysregulation.	Shen et al. (2024)
Mouse	SOD1-G93A	10X Genomics	Single nuclei isolation via ultracentrifugation	Primary	snRNA-seq pseudo time analyses across multiple CNS regions reveal that DAMs increase significantly during accelerated MN death. DAMs also display resting homeostatic marker and inhibitory checkpoint downregulation, driving increased neuroinflammation, phagocytosis and DAM hyperactivation.	Chen L. X. et al. (2024)
Mouse	*TARDBP*	10X Genomics	Single nuclei isolation via ultracentrifugation	Primary	snRNA-seq on mouse medial prefrontal cortex following selective TDP43 depletion revealed TDP43 loss induces severe GABAergic neuronal loss and calcium homeostasis dysregulation. Conversely, DAM marker genes were upregulated, suggesting TDP43 pathology may drive DAM hyperactivation.	Thapa et al. (2025)
Mouse	SOD1-G93A	10X Genomics	Single nuclei isolation via ultracentrifugation	Primary	snRNA-seq demonstrated that microglial accumulation near MNs drives DAM hyperactivation. Conversely, regulatory analyses identified CREB3 and ATF3 TFs drive MN transition into a “disease-associated motor neuron” (DM) state characterised by downregulated axonogenesis, synaptic transmission and increased apoptosis.	Gautier et al. (2026)
*Drosophila*	*C9orf72*-HRE	10X Genomics	Microdroplets	Primary	Longitudinal fly brain scRNA-seq across disease progression identified several vulnerable and resistant neuronal populations. Genetic cross-comparisons helped reveal neuronal vulnerability may be dictated by an initial transcriptional state, modulated by *Xbp1*.	Shen et al. (2025)

#### 1.1.1 *C9orf72* repeat expansion

A hexanucleotide GGGGCC (G_4_C_2_) repeat expansion (HRE) within the first intron of the *C9orf72* gene is by far the most common genetic risk factor for ALS, having been implicated in over 40% of fALS cases and 6% of sporadic cases (Sellier et al., 2024). Healthy individuals carry 2–23 G_4_C_2_ repeats, which can expand to hundreds or thousands of G_4_C_2_ repeats in ALS patients (DeJesus-Hernandez et al., 2011; Renton et al., 2011).

HRE negatively impact *C9orf72* gene expression, with haplo-insufficiency of *C9orf72* thought to be a contributory factor in ALS progression (Sellier et al., 2024). Although the precise function of C9orf72 remains poorly understood, recent studies have shown that C9orf72 regulates OXPHOS complex I assembly as well as stress granule (SG) formation via autophagy (Maharjan et al., 2017; Chitiprolu et al., 2018; Wang et al., 2021). Loss of *C9orf72* expression may therefore negatively impact both mitochondrial and SG homeostasis (Sellier et al., 2024).

Alongside loss of function (LoF), toxic gain of function mechanisms also contribute to *C9orf72*-ALS pathogenesis (Mizielinska et al., 2025). Aberrantly expanded G_4_C_2_ repeats are transcribed into three of the *C9orf72* characterised mRNA transcript variants (V2 and V3). In V1 and V3 the G_4_C_2_ repeats are located in the first intron, whilst in V2 they are within the promoter region (Figure 1) (Gijselinck et al., 2012; Gendron et al., 2013; Sellier et al., 2024; Mizielinska et al., 2025). The repeats are also transcribed in the antisense direction (Mizielinska et al., 2025). These sense and antisense transcripts can bind chromatin at promoter regions as well as form RNA foci, sequestering RNA-binding protein (RBP) and driving dysregulation of transcriptional and RNA metabolism (Figure 2A) (Fratta et al., 2012; Mizielinska et al., 2013; Haeusler et al., 2014; Su et al., 2014; Zhou et al., 2018; McEachin et al., 2020; Liu et al., 2026).

**Figure 1:**
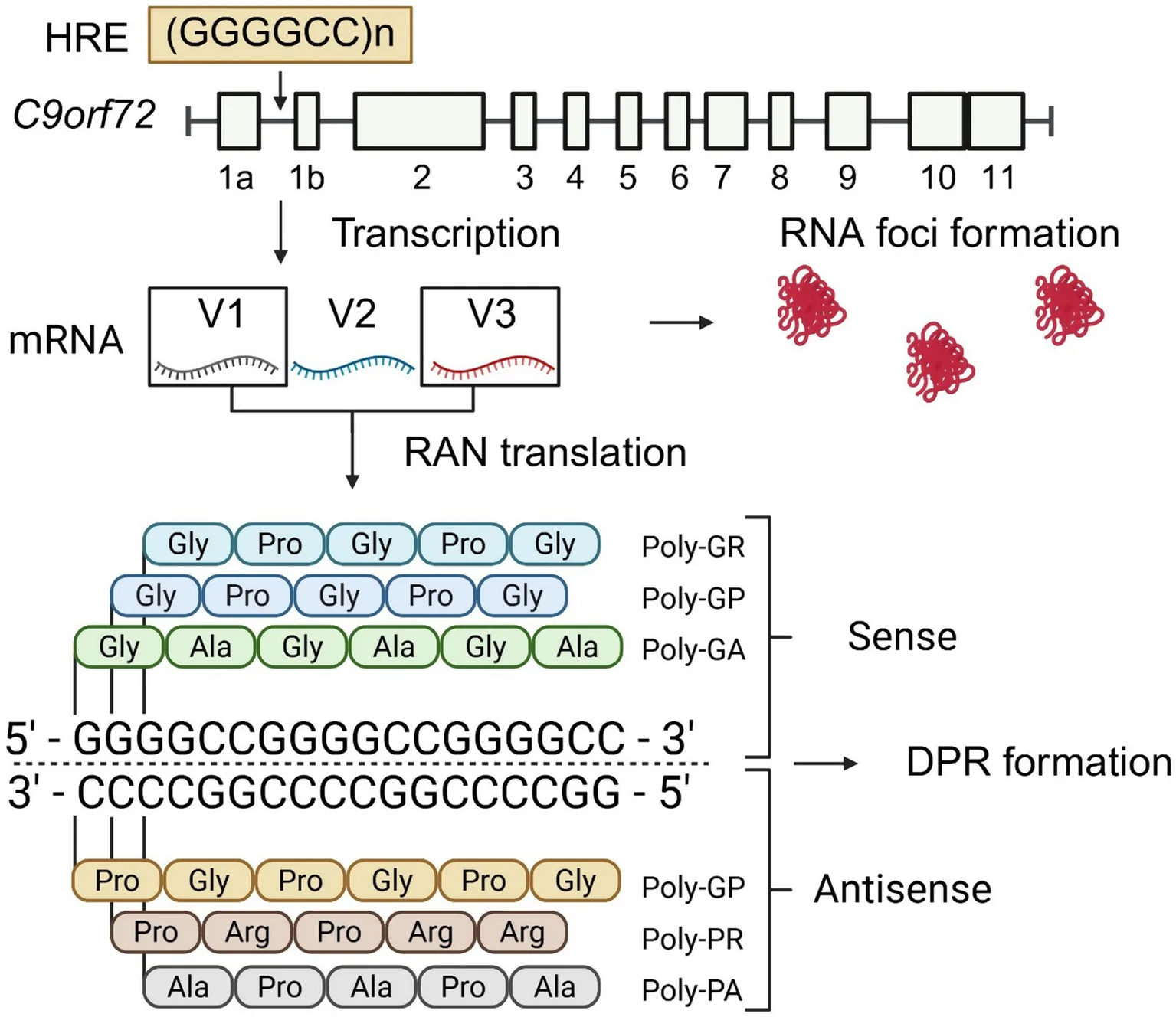
*C9orf72* DPR formation. Aberrantly expanded GGGGCC hexanucleotide repeats (HRE) in the first *C9orf72* intron are transcribed into three mRNA transcript variants (V1, V2 and V3) via cellular transcription machinery. These mRNAs can then go on to aggregate and form RNA foci within the cell. The V1 and V2 mRNAs can also undergo translation via non-canonical repeat-associated non-ATG-dependent (RAN) translation in both the sense and antisense direction. This results in the formation of toxic dipeptide repeat (DPR) species; glycine–alanine (Poly-GA), glycine-arginine (Poly-GR) and glycine-proline (Poly-GP) from the sense strand, and proline-alanine (Poly-PA), proline-arginine (Poly-PR) and Poly-GP from the antisense strand. *C9orf72* gene structure and DPR coding frames from (Balendra and Isaacs, 2018). Figure created using https://BioRender.com

**Figure 2:**
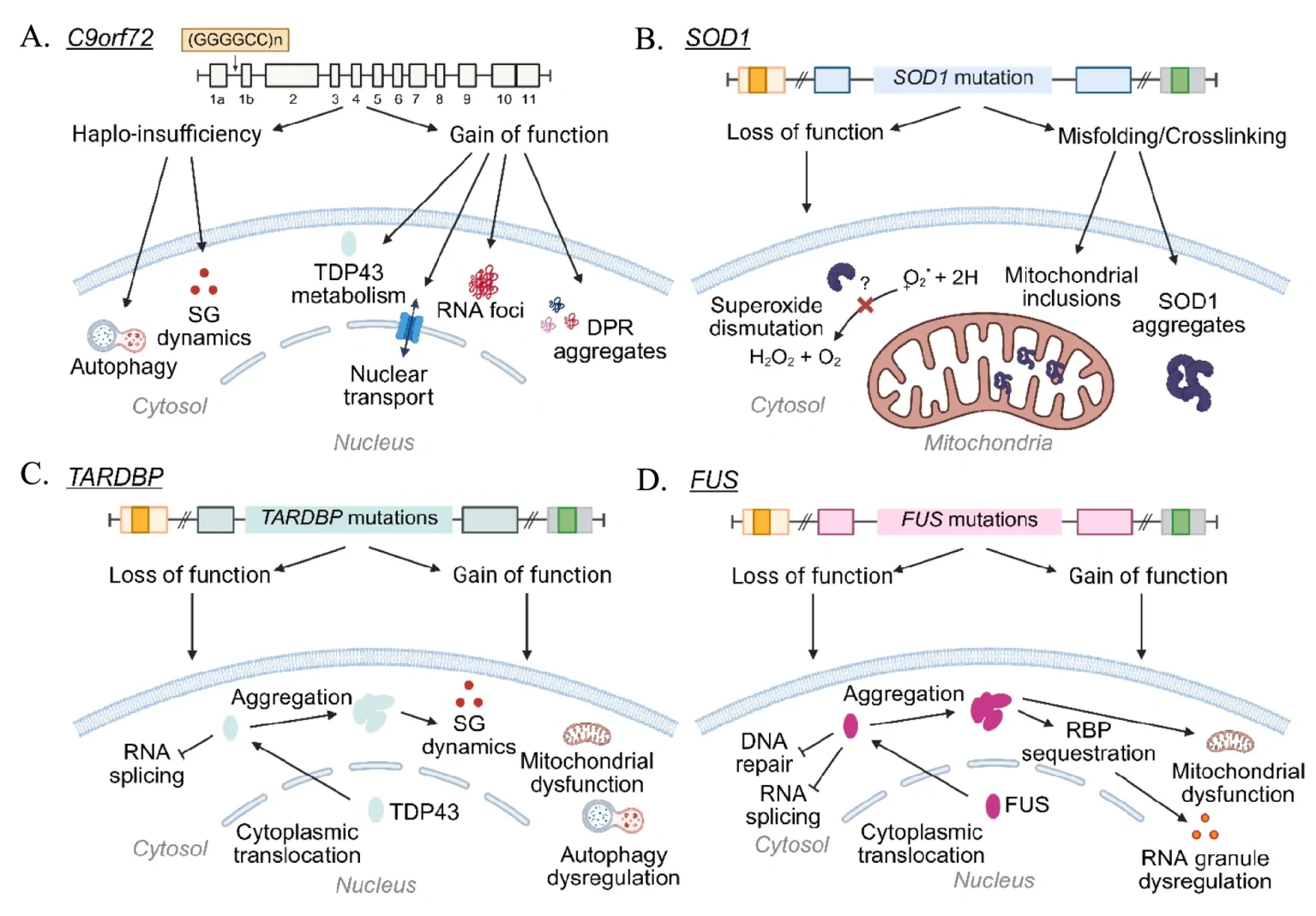
Genetic risk factors of ALS and their associated cellular pathology. **(A)** Hexanucleotide repeat expansions in *C9orf72* cause haplo-insufficiency, impacting autophagy, stress granule (SG) dynamics, nuclear transport and TDP43 metabolism. Toxic gain of function is associated with RNA foci formation and toxic dipeptide repeat (DPR) aggregation. **(B)** *SOD1* mutations result in abnormal misfolding and crosslinking, driving the formation of toxic cytoplasmic and mitochondrial SOD1 aggregates. **(C)** *TARDBP* mutations lead to loss of TDP43 nuclear localisation and RNA splicing activity. Mislocalised TDP43 is further prone to aggregation, impacting autophagy, SG dynamics and mitochondrial function. **(D)** *FUS* mutations cause a loss of FUS nuclear localisation, impairing DNA repair and RNA splicing pathways. Mislocalised FUS is subsequently prone to aggregation, sequestering RNA binding proteins (RBP), dysregulating RNA granule homeostasis and driving mitochondrial dysfunction. Figure created using https://BioRender.com.

Aside from RNA-mediated toxicity, *C9orf72*-HRE also induce toxicity through dipeptide repeat (DPR) proteins (Sellier et al., 2024). Sense and antisense transcripts are translated into DPRs via non-canonical repeat-associated non-ATG-dependent (RAN) translation (Figure 1) (Gendron et al., 2013). This results in the formation of 5 DPR species: glycine–alanine (Poly-GA), glycine-proline (Poly-GP), glycine-arginine (Poly-GR), proline-alanine (Poly-PA) and proline-arginine (Poly-PR) (Figure 1) (Gendron et al., 2013; Mori et al., 2013; Zu et al., 2013). DPRs form both cytoplasmic and nuclear inclusions, containing p62 but not TDP43 (Figure 2A) (Al-Sarraj et al., 2011; Mann et al., 2013). *In vivo* work has shown that DPRs may be driving toxicity, affecting several critical cellular processes (Mizielinska et al., 2014; Wen et al., 2014; Freibaum et al., 2015; O'Rourke et al., 2015; Jiang et al., 2026). For example, Poly-GR, Poly-PR and Poly-GA disrupt both RNA and protein nucleocytoplasmic transport by impairing the nucleocytoplasmic transporters RanGAP and GTPase Ran and both Poly-PR and Poly-GR can bind ribosomal subunits and spliceosome components, blocking SG dynamics and protein translation (Figure 2A) (O'Rourke et al., 2015; Tao et al., 2015; Loveland et al., 2022; Ryan et al., 2022). DPRs are also strongly linked to TDP43 pathology, driving TDP43 nuclear clearance and cytoplasmic aggregation (Figure 2A) (Cook et al., 2020; Agnihotri et al., 2025). However, the specific details of how pathways affected by aberrant RNA and DPRs drive ALS still remain poorly understood, making an improved mechanistic understanding critical for successful therapeutic development (Mizielinska et al., 2025).

#### 1.1.2 *SOD1* mutations

Mutations in the *SOD1* gene make it the second most common genetic risk factor for fALS, causing 21.1% of fALS cases and 2.3% of sporadic cases (Berdyński et al., 2022). *SOD1* encodes an antioxidant enzyme responsible for the dismutation of the superoxide anions, generated mostly by mitochondrial redox reactions, to hydrogen peroxide (Bunton-Stasyshyn et al., 2014). Over 200 *SOD1* ALS-associated mutations have been found, mostly missense mutations in coding exons (Figure 3; Table 1) (Rosen et al., 1993; Namboori et al., 2021; Benatar et al., 2025) which alter the charge of the protein, severely compromising its normally stable homodimer architecture (Benatar et al., 2025). This destabilisation facilitates the formation of abnormal disulphide bonds, protein misfolding and aggregation and leads to the accumulation of mutant SOD1 in the mitochondrial intermembrane space (IMS) in transgenic ALS rodent MN (Figure 2B) (Jaarsma et al., 2001; Ahtoniemi et al., 2008; Tafuri et al., 2015;DiDonato et al., 2003; Forsberg et al., 2010; Rakhit et al., 2007). Interestingly, though loss of SOD1 antioxidant activity was initially considered the primary causative agent of *SOD1*-ALS, activity levels of several SOD1 mutant variants remain normal and the successful trial of the Tofersen *SOD1* mRNA degrading antisense oligonucleotide (ASO) points to a gain of function mechanism (Figure 2B) (Keskin et al., 2017; Huang et al., 2024; Benatar et al., 2025; McGuigan and Blair, 2025).

**Figure 3:**
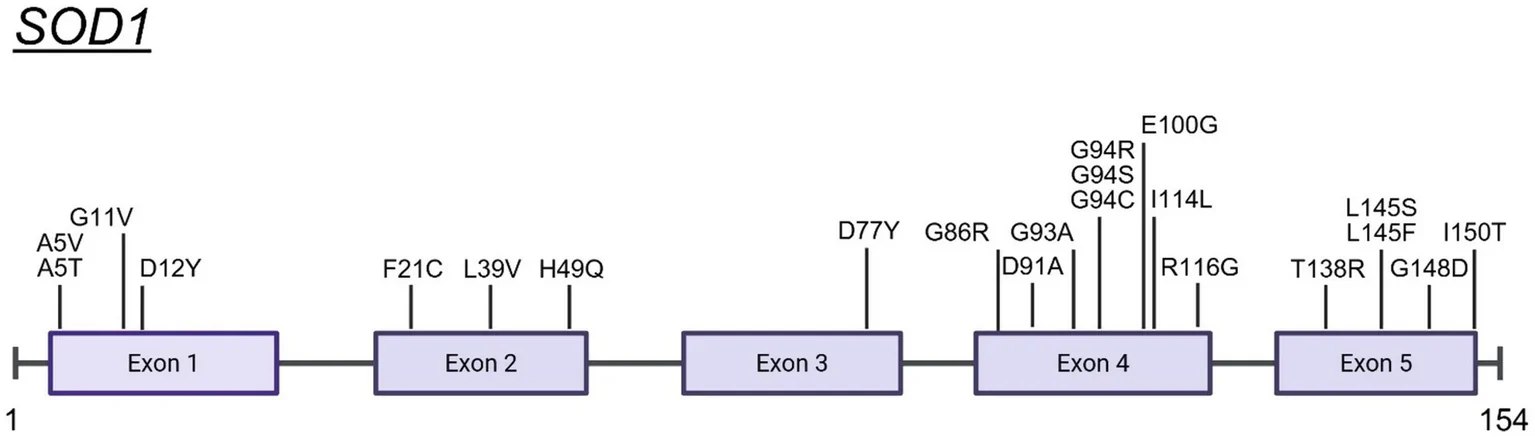
*SOD1* ALS-associated missense point mutations. Schematic representation of the *SOD1* gene indicating missense point mutations linked to ALS in the SOD1 protein related to the studies discussed in this Review. Missense point mutations are placed according to their exon localisation. Numbers indicate amino acid length. Data compiled from (Rosen et al., 1993; Benatar et al., 2025), domains from https://www.uniprot.org/. Figure created using https://BioRender.com.

Mutant SOD1-G93A drives increased oxidative stress (OS) in mice, contributing to mitochondrial depolarization and reducing mitochondrial responsiveness to OS with age and has been shown to induce ER stress and alterations in ER morphology (Zhao et al., 2022; Guo et al., 2024). Despite these advances, the precise mechanisms remain poorly understood and it remains unclear to what extent misfolded wildtype SOD1 contributes to disease progression in ALS patients lacking *SOD1* mutations, with conflicting reports surrounding the presence of SOD1 inclusions in these patients (Wang et al., 2024; Benatar et al., 2025).

#### 1.1.3 *TARDBP* mutations

*TARDBP* gene mutations are also linked to fALS onset, with some 60 different mutations identified so far in over 3.3% of fALS cases and 0.5% sporadic cases (Figure 4; Table 1) (Chen et al., 2021). *TARDBP* encodes the primarily nuclear protein TDP43, which regulates RNA transport, transcription and splicing (Lagier-Tourenne et al., 2010). It inhibits cryptic exon (CE) inclusion and represses transcription of several genes (including *TARDBP* and *SP10*) through binding TG-rich elements in promoters (Ou et al., 1995; Acharya et al., 2006; Abhyankar et al., 2007; Ma et al., 2022). In the cytoplasm it is involved in cytoplasmic RNA granule formation to transport target mRNAs to distal neuronal compartments (Alami et al., 2014).

**Figure 4:**
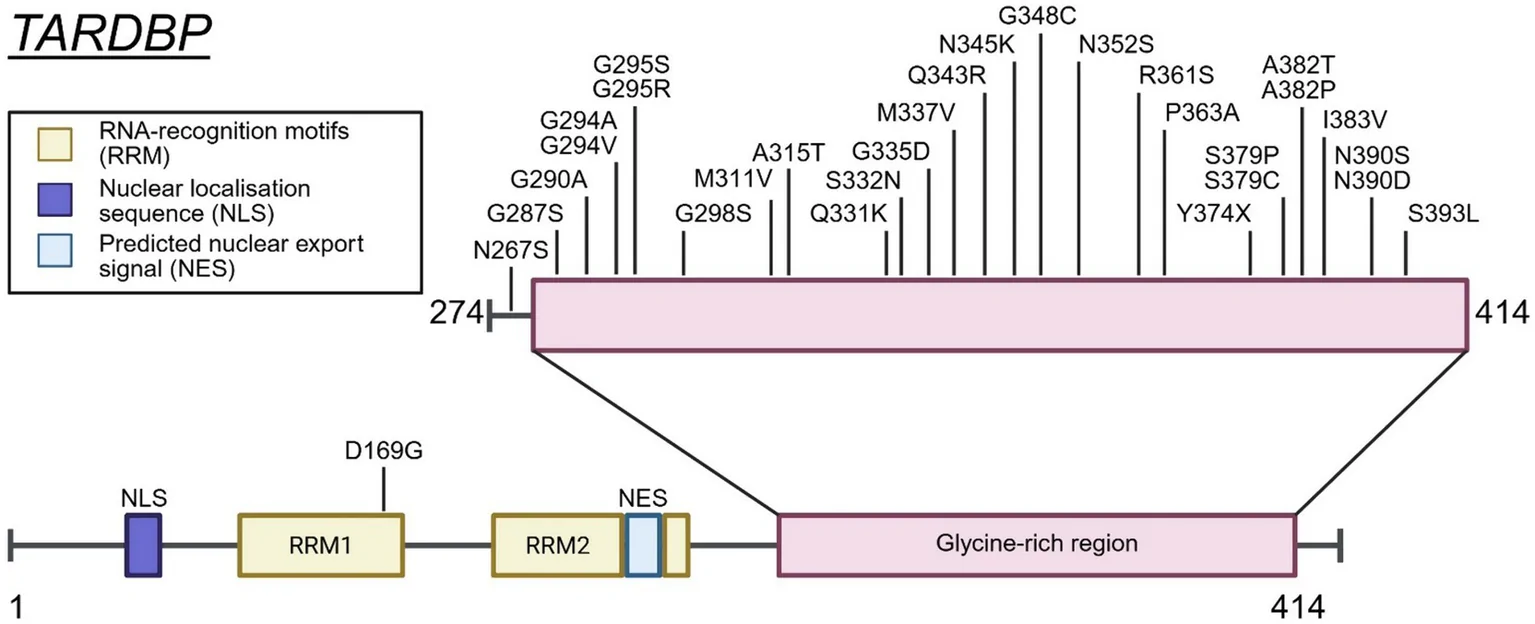
*TARDBP* ALS-associated missense point mutations. Schematic representation of the *TARDBP* gene indicating missense point mutations linked to ALS in the TDP43 protein related to the studies discussed in this Review. Missense point mutations are placed according to their exon localisation. Numbers indicate amino acid length. Data compiled from (Lagier-Tourenne and Cleveland, 2009), domains from https://www.uniprot.org/. Figure created using https://BioRender.com.

Disease linked mutations can alter post-translational TDP43 phosphorylation and ubiquitination patterns, causing a loss of nuclear TDP43 and increased cytoplasmic localization (Figure 2C) (Arai et al., 2006; Neumann et al., 2006; Inukai et al., 2008). TDP43 mis-localization then drives RNA splicing dysregulation, leading to CE inclusion within genes, causing frameshifts and premature truncation across several proteins critical for neuronal function, including *STMN2* and *UNC13A* (Torres et al., 2018; Klim et al., 2019; Melamed et al., 2019; Brown et al., 2022; Ma et al., 2022; Koike et al., 2023).

Mislocalised TDP43 has also been linked to mitochondrial dysfunction, SG formation and autophagy dysregulation (Liu-Yesucevitz et al., 2010; Stribl et al., 2014; Xia et al., 2016). Importantly, TDP43 aggregates have been found in over 97% of ALS cases and are a pathological hallmark of ALS, suggesting TDP43 is a major disease driver (Swarup et al., 2011; Ling et al., 2013). TDP43 appears to contribute to ALS progression through both loss of nuclear function and cytoplasmic toxic gain of function linked to aggregation (Figure 2C) (Bradford and Rodgers, 2024). Even though the specific mechanisms driving neurodegeneration are not fully understood, near ubiquitous TDP pathology presence across ALS cases makes TDP43 modulation a highly attractive treatment target (Swarup et al., 2011; Ling et al., 2013; Bradford and Rodgers, 2024).

#### 1.1.4 *FUS* mutations

Mutations in the *FUS* gene have also been linked to fALS onset, particularly within juvenile patients, with *FUS* mutations identified in 2.8% European and 6.4% Asian fALS cases (Figure 5) (Zou et al., 2017; Moens et al., 2025). *FUS* mutations have also been identified with a lower frequency in sporadic cases, with an incidence of 0.3% in European and 0.9% in Asian cohorts (Zou et al., 2017). The *FUS* gene encodes a critical nuclear-localised RBP involved in genomic stability, DNA repair, translation and transcription (Lagier-Tourenne et al., 2010; Chen et al., 2019).

**Figure 5:**
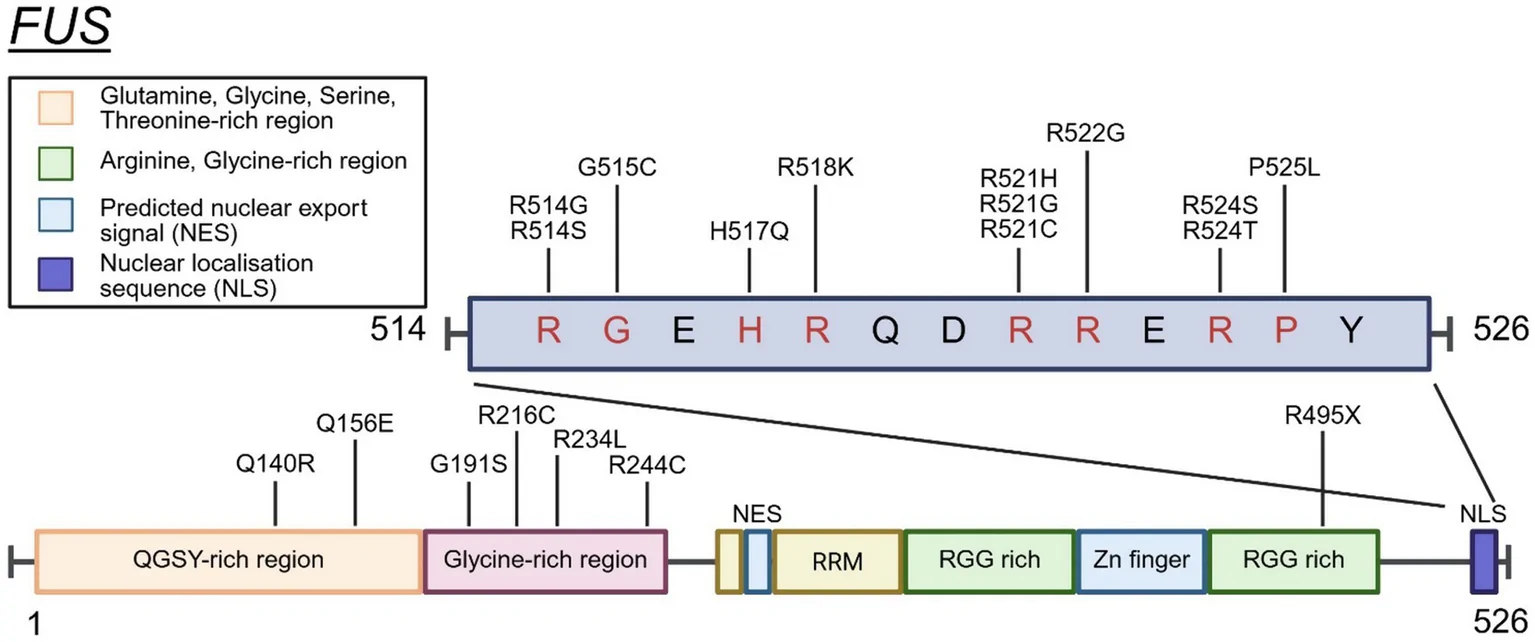
*FUS* ALS-associated missense point mutations. Schematic representation of the *FUS* gene indicating missense point mutations linked to ALS in the FUS protein related to the studies discussed in this Review. Missense point mutations are placed according to their exon localisation. Numbers indicate amino acid length. Data compiled from (Xiao et al., 2024), domains from https://www.uniprot.org/. Figure created using https://BioRender.com.

Disease-associated mutations within the FUS C-terminal region nuclear localisation sequence (NLS) inhibit nuclear retainment and cause aberrant FUS localization to the cytoplasm, leading to a dominant LoF phenotype often associated with defective RNA splicing (Figure 2D) (Nakaya and Maragkakis, 2018). Mutant FUS-P525L expression sequesters snRNA into cytoplasmic aggregates, driving intron splicing defects and impairs DNA damage response pathways (Reber et al., 2016; Naumann et al., 2018). DNA damage itself then enhances FUS mis-localisation via DNA-PK activation, driving a vicious cycle of FUS nuclear depletion and neurodegeneration in hiPSC-MN lines (Naumann et al., 2018).

Cytoplasmic FUS is also linked to RNA-granule solidification, RBP sequestration and phase transition acceleration (Figure 2D) (Qiu et al., 2014; Takanashi and Yamaguchi, 2014; Patel et al., 2015; Scekic-Zahirovic et al., 2016; Birsa et al., 2021). Mutant FUS also shows greater mRNA affinity, which can lead to ribosomal stalling and premature translation termination (Kamelgarn et al., 2018). Recent FUS-R495X and FUS-P525L hiPSC-MN studies have also pointed to a link with mitochondrial dysfunction (Bradford and Rodgers, 2024; Schweingruber et al., 2025).

### 1.2 Models of ALS

#### 1.2.1 *In vivo* models of ALS

##### 1.2.1.1 *Drosophila* models

The fruit fly, *Drosophila melanogaster,* is one of the most widely used model systems in research, with over a 100 years of use (Bellen et al., 2010). Its simplicity, ease of genetic manipulation and vast availability of a genetic toolkit has made *Drosophila* a widely used model for several neurodegenerative diseases, including ALS (Hegde and Srivastava, 2022).

The translational relevance of *Drosophila* is underscored by its high genetic homology to humans, with over 77% of human disease genes having fly orthologues (Hegde and Srivastava, 2022). Crucially, *Drosophila’s* compact brain architecture allows researchers to measure ageing and peripheral tissue influence across the entire brain simultaneously (Omelyanchuk et al., 2015).

Clear phenotypic readouts are another major advantage of using flies, with an abundance of highly sensitive, easily scorable phenotypes that can be used to characterise neurotoxicity. For example, researchers can evaluate lifespan, perform climbing assays to measure upward mobility, or assess eye morphology, which provides a direct, immediately visible readout of neurodegeneration as the fly eye is largely neuronal in origin (Jackson, 2008; Madabattula et al., 2015). All these assays are easy and quick enough to be usable at scale, allowing flies to be used for screening large numbers of genes or drugs for rescue of phenotypes.

*Drosophila* models of ALS genetic risk factors involve the over-expression of human disease-associated genes in neurons. *SOD1* mutations were amongst the first to be explored in flies, demonstrating that over-expression of human mutant SOD1 in neurons. (Watson et al., 2008) recapitulated many ALS phenotypes, including locomotor defects, shortened lifespan and neurodegeneration (Hegde and Srivastava, 2022). Similarly, genetic knock-in models of SOD1-G85R/H48R/H71Y mutations recapitulated similar lifespan and motor function defects (Şahin et al., 2017; Hegde and Srivastava, 2022).

Overexpression of human mutant TDP43, FUS and *C9orf72*-HRE in fly neurons also results in neuron loss, disease-type specific proteinopathy and lifespan shortening (Li et al., 2010; Mizielinska et al., 2014; Zhang et al., 2015; Fortuna et al., 2021). These models have also been instrumental in identifying several novel modulators of disease (Liguori et al., 2021).

Flies have also been used to successfully screen genetic and pharmacological modifiers of disease (Lenz et al., 2013). A drug screen in flies expressing the mutant TDP43-G348C protein revealed that the FDA-approved drug Fingolimod improves locomotor defects and reduces pupal lethality, highlighting the value of *Drosophila* ALS models in drug repurposing and a genetic screen in a *C9orf72*-HRE model identified nucleocytoplasmic transport as a key driver of toxicity (Zhang et al., 2015; Lo Piccolo et al., 2023).

Together, the availability of a vast genetic toolkit, combined with simple, scalable phenotypic read-outs and brief lifespans makes *Drosophila* a powerful ALS model system which allows researchers to monitor disease development disease development across the whole lifespan and carry out modifier screens, facilitating the rapid identification of relevant pathways, which have subsequently been confirmed in human neurons (Kim and Taylor, 2017; Layalle et al., 2021; Bonini, 2022; Nitta and Sugie, 2022; Todd and Petrucelli, 2022; Shen et al., 2025). Despite this, flies have some limitations as ALS models, including drastic differences in brain anatomy and the absence of more complex vertebrate systems that may influence neurodegeneration, such as adaptive immunity and brain inflammation (Hegde and Srivastava, 2022).

##### 1.2.1.2 Mouse models

On the other hand, mouse ALS models are more closely related to humans whilst maintaining genetic tractability (Zhou et al., 2024). This provides a vital bridge to human biology and excellent translational relevance. They display high similarities in genetics (over 90% similarity), brain architecture, mammalian physiology, axon myelination and hormones, as well as the presence of a more developed immune system (Jucker, 2010; Breschi et al., 2017; Fisher and Bannerman, 2019; Todd and Petrucelli, 2022). This offers distinct advantages over *Drosophila*, making mice powerful tools for studying mammalian physiology (Jucker, 2010; Breschi et al., 2017; Fisher and Bannerman, 2019; Todd and Petrucelli, 2022).

A number of ALS mouse models for key mutations in *SOD1*, *TDP43*, *FUS* and *C9orf72* have been engineered. Whilst early models relied on mutant over-expression (e.g., SOD1-G93A, TDP43-A315T, FUS-R521C and *C9orf72*-HRE) modern approaches utilise endogenous knock-ins (e.g., SOD1-D83G, TDP43-N390D, FUS Delta 14) and conditional knock-outs or complex combinations, such as *TARDBP* floxed constructs or combining C9-BAC mice with *C9orf72* knockouts to more accurately replicate disease (Gurney et al., 1994; Wegorzewska et al., 2009; Joyce et al., 2015; Devoy et al., 2017; Donde et al., 2020; Huang et al., 2020; Zhang et al., 2020; Todd and Petrucelli, 2022; Ma et al., 2023).

Phenotypic readouts across these diverse models successfully capture both the organism-level symptoms and cellular pathologies of ALS (Todd and Petrucelli, 2022). Most models exhibit locomotor defects, paralysis and shortened lifespans together with progressive MN degeneration. Whilst recapitulating cytoplasmic protein aggregates was challenging in early *TDP43* and *FUS* models, newer models demonstrate robust intracellular mutant-associated hallmarks (Scekic-Zahirovic et al., 2016; Shiihashi et al., 2016; Todd and Petrucelli, 2022). For instance, advanced *TDP43* and *C9orf72* models usually exhibit TDP43-positive inclusions, RNA foci, DPR formation and cortical hyperexcitability, effectively mirroring human physiological disease progression and underscoring their translational relevance progression (Chew et al., 2019; Cook et al., 2020; Vucic et al., 2021; Milioto et al., 2024).

Despite harbouring distinct genetic mutations that drive unique initial pathologies, these models ultimately converge on similar disease states: similar cellular pathways are dysregulated across ALS mouse models, such as RNA metabolism, mitochondrial functionality and protein homeostasis, all of which have been implicated in ALS pathogenesis (Lutz, 2018; Todd and Petrucelli, 2022).

Together, transgenic mouse ALS models provide a powerful platform to help understand the mechanistic underpinnings of fALS, as well as for therapeutics testing (Lutz, 2018). Their strengths include their high translational relevance, underpinned by strong neurological, physiological and genetic similarities to humans (Jucker, 2010; Breschi et al., 2017; Fisher and Bannerman, 2019; Todd and Petrucelli, 2022). However, the long lifespans and high costs limits throughput and long-term therapeutic assessments (Zhou et al., 2024).

#### 1.2.2 *In vitro* ALS models

##### 1.2.2.1 Human-derived induced pluripotent stem cells (hiPSC) models

Despite the many advantages of mouse ALS models, the lack of a human biological context remains a limitation that can hinder accurate disease recapitulation and translation (Giacomelli et al., 2022). To overcome these challenges, human-induced pluripotent stem cell (hiPSC) models have been developed to study ALS pathologies *in vitro* (Giacomelli et al., 2022).

Since their introduction by Takahashi et al. (2007), iPSCs derived from patient somatic cells, such as fibroblasts or peripheral blood cells, have become one of the most widely used *in vitro* model systems (Ferraiuolo and Maragakis, 2021). Because they can be differentiated into all key lineages of the nervous system, including neurons and microglia, iPSCs allow researchers to accurately model cell-type-specific disease mechanisms (Takahashi et al., 2007; Ferraiuolo and Maragakis, 2021; Dawoody Nejad and Pioro, 2025).

Large repositories of hiPSC lines from ALS patients and controls have been generated, differentiated into MN and other relevant cell types like microglia, helping model the full range of ALS-relevant human cell types (Workman et al., 2023). However these lines can display significant individual variability (Workman et al., 2023). To overcome this, isogenic lines have been generated using CRISPR/Cas9 gene-editing. This is achieved either by introducing ALS-associated mutations into healthy control lines or by correcting mutations in patient-derived lines to create true isogenic controls (Ababneh et al., 2020; Schweingruber et al., 2025).

hiPSC-derived MN display several disease phenotypes, at least to some extent. MN with *FUS* and *TARDBP* ALS-mutations display SG dynamics defects, whilst models expressing *C9orf72*-HRE show DPR and RNA-foci pathologies, along with inhibited nucleocytoplasmic transport, synaptic trafficking defects, sensitivity to glutamate toxicity and increased calcium permeability (Donnelly et al., 2013; Lagier-Tourenne et al., 2013; Sareen et al., 2013; Freibaum et al., 2015; Dafinca et al., 2016; Selvaraj et al., 2018; Fang et al., 2019; Coneys et al., 2025). Similarly, although less extensively studied, *SOD1* mutant hiPSC-MNs display altered axonal and neurofilament dynamics (Kim et al., 2020; Giacomelli et al., 2022).

Given their genetic tractability and scalability, iPSC-derived MN have been especially useful as disease-relevant models for compound screens, leading to the discovery of small-molecules that suppress TDP43 recruitment to SGs and increase neurofilament light chain (NF-L) levels (Fang et al., 2019; Hölbling et al., 2025; Sahin et al., 2026).

iPSCs have been particularly useful in modelling the early dynamics of ALS pathogenic molecular mechanisms (Ardhanareeswaran et al., 2017). These models are crucial for complementing studies that use post-mortem patient tissue samples, which can only provide information about end state disease (Giacomelli et al., 2022). However, limitations remain, including the inability to capture the complex neural tissue environment and the non-cell-autonomous interactions that are present *in vivo* (Ardhanareeswaran et al., 2017). Importantly, many of the reprogramming processes used in hiPSC-MN differentiation result in embryonic-like cells that lack age-related traits (Venkataraman et al., 2022; Calvo et al., 2025).

#### 1.3 ScRNA seq

The first true description of scRNA-seq analysis was in 2009 by Tang et al., on single mouse blastomeres, cementing scRNA-seq as a cutting-edge technique to revolutionise our ability to monitor transcriptional regulation in multicellular systems (Tang et al., 2009). In comparison to bulk RNA-sequencing technologies, scRNA-seq focuses on analysing transcriptomes from individual cells within heterogeneous populations, allowing the identification of distinct cellular subtypes and expression patterns obscured in bulk analyses (Hwang et al., 2018; Molla Desta and Birhanu, 2025).

##### 1.3.3 ScRNA-seq pipeline

ScRNA-seq, firstly, requires the isolation of individual cells from heterogenous tissues (Sant et al., 2023), either by flow-activated cell sorting (FACS) or microfluidics (Figure 6) (Hwang et al., 2018). FACS sorts encapsulated cells by applying electromagnetic charges to fluorescently labelled cells and deflecting them into a separate tube (Julius et al., 1972; Hwang et al., 2018). This allows for efficient cell capture, but requires large starting volumes (>10,000 cells) (Hwang et al., 2018).

**Figure 6:**
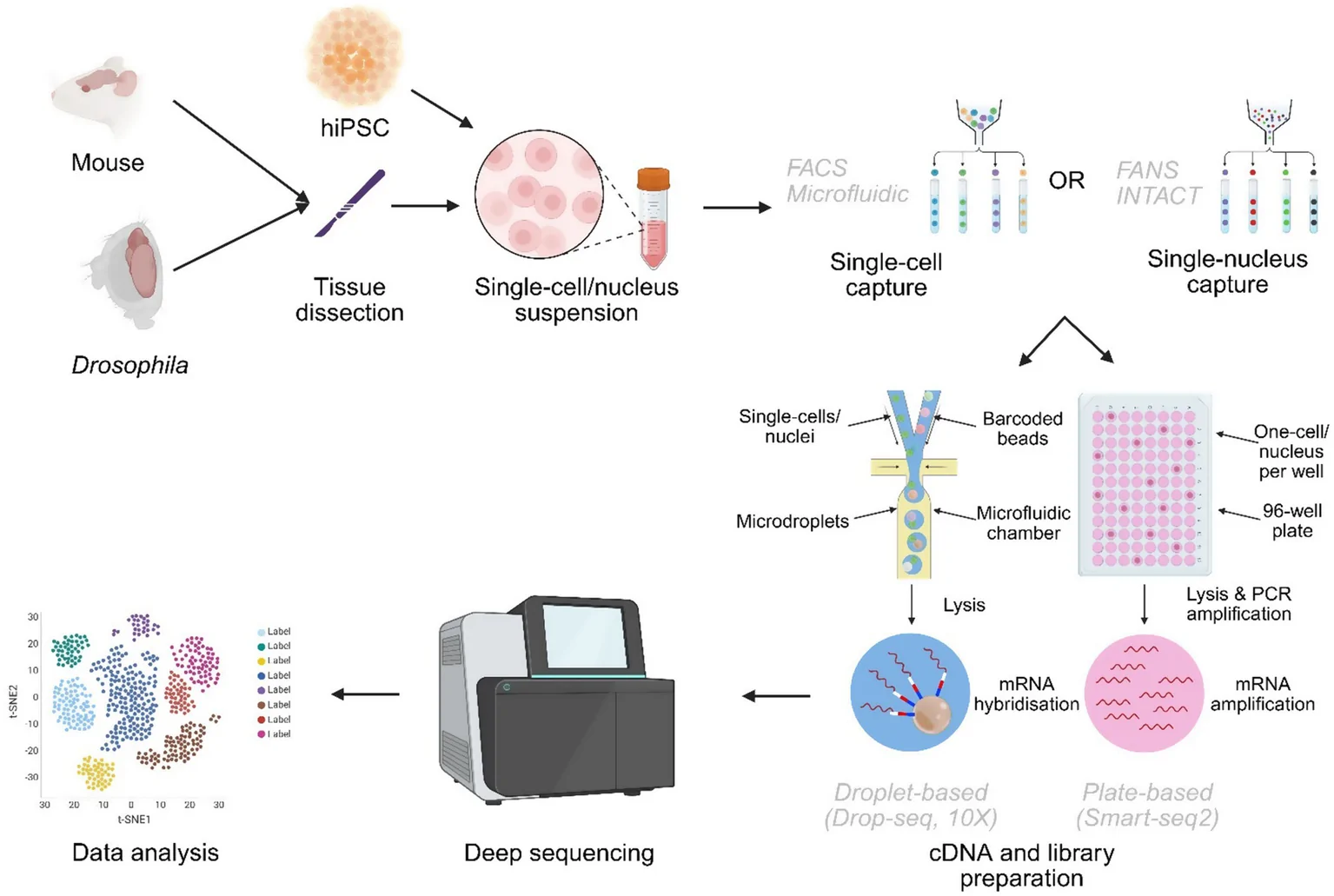
ScRNA-seq/snRNA-seq workflow. Dissected tissue samples from the relevant tissue are converted into a single-cell/nucleus suspension, with hiPSC being suspended directly. Single cells are then captured using either flow-activated cell sorting (FACS) or microfluidics. Meanwhile single nuclei can be captured using fluorescence-activated nuclei sorting (FANS) or isolation of nuclei tagged in specific cell types (INTACT). Following single-cell capture, cellular mRNA is extracted, reverse transcribed to cDNA and barcoded via plate-based (Smart-seq2) or droplet-based (Drop-seq, 10X Genomics) techniques. Plate-based techniques lyse, amplify and sequence single cells in individual wells on a 96-well plate. Droplet-based techniques suspend cells in microdroplets containing barcoded beads and lysis enzymes, with mRNA hybridisation to barcodes facilitating transcript indexing and cell identification. Generated cDNA libraries are then sequenced using deep sequencing platforms such as Illumina, with downstream computational data analysis facilitating cluster and gene expression mapping. Figure created using https://BioRender.com.

By contrast, microfluidic devices use nanolitre-sized sample volumes to sort single cells via a system of valves and switches, utilising multiple sorting channels to increase throughput, reducing false positives and increasing sorting efficiency (Shields et al., 2015; Yousuff et al., 2017; Jin et al., 2021). Importantly, microdroplet-based microfluidics require lower volumes than standard chambers by isolating cells in a continuous oil phase as monodispersed aqueous droplets, increasing throughput and reducing costs (Utada et al., 2005; Islam et al., 2014; Hwang et al., 2018). These small sample requirements, coupled with low costs, have significantly contributed to their expansion (DeLaughter, 2018; Hwang et al., 2018).

Once single cells have been isolated, cDNA libraries are generated where RNA molecules are barcoded to facilitate subsequent sequencing and analysis (Pozojevic and Spielmann, 2023; Sant et al., 2023; Molla Desta and Birhanu, 2025). Plate-based techniques, like Smart-seq2, isolate cells into individual wells where RNA is converted into full-length cDNAs, fragmented and labelled with dual-index barcodes (Figure 6) (Trombetta et al., 2014; Li, 2021). Smart-seq2 provides excellent sequencing depth and sensitivity, picking up alternative splicing events and lowly expressed transcripts, but struggles with low throughput and PCR amplification bias (Ramsköld et al., 2012; Hwang et al., 2018; Li, 2021). Conversely, droplet-based techniques such as 10X Genomics encapsulate single cells together with beads carrying oligo-dT primers containing a cell specific barcode and a 4–8 base pair Unique Molecular Identifier (UMI) to index individual transcripts (Figure 6) (Hwang et al., 2018; Li, 2021). This bead-based approach dramatically increases throughput by allowing thousands of cells to be processed simultaneously in a single tube, whilst the UMIs effectively neutralise PCR bias to ensure accurate transcript counting (Li, 2021).

Following cDNA library sequencing, standard computational analysis pipelines involve quality control, normalisation, integration, clustering, differential gene expression (DGE) and visualisation (Hwang et al., 2018; Molla Desta and Birhanu, 2025). Initial quality control steps use tools like Scanpy or Seurat to filter out compromised or stressed cells based on thresholds such as mitochondrial gene proportions and UMI counts (Osorio and Cai, 2021; Pereira et al., 2021; Bishara et al., 2022; Molla Desta and Birhanu, 2025). To resolve technical variability, integration tools like Harmony and BBKNN mitigate batch effects, enabling the accurate clustering of cells into specific populations based on their expression profiles (Zappia et al., 2018; Korsunsky et al., 2019; Polański et al., 2020). Subsequent DGE analysis uncovers dynamic cellular states, which are ultimately displayed using dimensionality reduction algorithms like UMAP and t-SNE to convert complex, high-dimensional data into accessible 2D or 3D visual representations (Xiang et al., 2021; Das et al., 2022). Common analytical pitfalls and relevant mitigation steps are summarised in Table 3.

**Table 3:** Summary of common analytical challenges in scRNA-seq/snRNA-seq datasets and corresponding mitigation strategies.

Analytical challenge	Mitigation strategy	Reference
Low-quality cells	Filter cells with low UMI numbers and high percentage of mitochondrial genes (Seurat, FastQC, scCatalyzer)	Nesari et al. (2026)
Cell-type mis-annotation	Automated cell annotation utilising marker gene database-, supervision classification- and correlation-based methods (CellAssign, SingleR, CaSTLe)	Pasquini et al. (2021)
Doublets – two or more cells/nuclei captured together and sequenced as one	Utilise computational doublet identification packages to filter out doublets (DoubletFinder, cxds, bcds)	Xi and Li (2021)
Batch-effects	Normalise between different batches using integration tools (Harmony, BBKN).	Korsunsky et al. (2019); Polański et al. (2020)
Visualisation	Dimensionality reduction methods to visualise cell clusters (PCA, UMAP, t-SNE)	Xiang et al. (2021)

Whilst scRNA-seq provides invaluable insights into cellular heterogeneity, its application is severely limited in notoriously difficult-to-dissociate tissues like the brain. This challenge is compounded when using human patient samples, which are typically post-mortem and frozen. In frozen ALS brain samples, whole-cell isolation is particularly difficult; the mechanical stress of dissociation techniques often damages fragile, highly interconnected neurons, causing cell-type isolation bias, gene expression artefacts and the loss of distal compartment mRNA (Calvo et al., 2020; Armand et al., 2021; Jovic et al., 2022). Furthermore, the freeze–thaw cycles required for sample preparation compromise cell membranes, releasing ambient RNA into the suspension (Jiang et al., 2023). This leads to transcript cross-contamination across cell populations, making it practically impossible to isolate intact single-cell transcriptomes and confounding downstream scRNA-seq analysis (Yang et al., 2020; Jiang et al., 2023).

By contrast, nuclei are much easier to isolate from complex nervous tissues because the nuclear double-membrane is highly resistant to freeze–thaw damage (Nott et al., 2021; Jiang et al., 2023). This robustness allows researchers to accurately extract intact nuclei from frozen patient tissue (Jiang et al., 2023). Because only a small amount of nuclear mRNA is required to assess the cellular transcriptional state, nuclei serve as a superior source of single-cell mRNA in frozen samples, making snRNA-seq a powerful alternative (Jovic et al., 2022; Jiang et al., 2023). However, unlike scRNA-seq, snRNA-seq only profiles the nuclear transcriptome, meaning key cytoplasmic processes, such as RNA metabolism, may be missed (Jovic et al., 2022).

During snRNA-seq, nuclei are extracted via ultracentrifugation and isolated using either fluorescence-activated nuclei sorting (FANS) or isolation of nuclei tagged in specific cell types (INTACT) (Chongtham et al., 2021; Nott et al., 2021). FANS uses fluorescent antibodies for highly targeted sorting but suffers from slow processing, high costs and potential nuclear damage due to hydrodynamic stress (Chongtham et al., 2021). Conversely, INTACT captures nuclei rapidly and consistently via anti-tag magnetic beads (Handley et al., 2015). Researchers must weigh these trade-offs, as INTACT’s speed and consistency come at the cost of the higher isolation accuracy provided by FANS (Chongtham et al., 2021). Once the nuclei are isolated, cDNA library preparation and subsequent sequencing and analysis steps are the same as for scRNA seq.

##### 1.4 Single cell analysis of ALS patients

Single-cell and single-nucleus RNA sequencing have revolutionised our understanding of cell-type-specific transcriptional responses to environment and disease, uncovering cellular nuances previously hidden in bulk studies. In the brain, a dense assembly of over 3,000 highly intermixed neuronal and glial subtypes, single-cell technologies are essential to uncover true cell type specific signals (Armand et al., 2021; Soorajkumar et al., 2025). The Brain Cell Atlas has successfully mapped novel neuronal subtypes, whilst scRNA-seq mapping of the spinal cord has revealed how some known ALS risk factors are uniquely enriched in adult MNs, possibly going some way to explaining their unique vulnerability to disease (Yadav et al., 2023; Chen X. et al., 2024).

More targeted studies have been able to pinpoint even further this cell type specific vulnerability. Recent transcriptomic analyses of post-mortem ALS tissue highlight intrinsic pathological cascades driving differential vulnerability in extra-telencephalic (ET) corticospinal MNs (CSMNs), notably layer 5 (L5) upper MNs (Gittings et al., 2023; Limone et al., 2024; Pineda et al., 2024). Across these susceptible populations there is widespread dysregulation in energy homeostasis, mitochondrial respiration and proteostasis, with upregulated proteasomal subunits and heat-shock chaperonins (Limone et al., 2024; Pineda et al., 2024). RNA/tRNA metabolism is also impaired, for example, in *C9orf72*-ALS, TDP43 pathology in L5 neurons drives nonsense-mediated decay (NMD) and cryptic exon accumulation, disrupting ribosomal and transcriptional networks (Gittings et al., 2023). Gene set enrichment analysis (GSEA) further links L5 susceptibility to impaired ciliary axoneme maintenance; given the role of primary cilia in axonal guidance, long-range projection neurons are particularly vulnerable to these functional deficits during disease progression (Pineda et al., 2024).

Beyond intrinsic defects, sc/snRNA-seq emphasises cell non-autonomous drivers of ALS, specifically neuroinflammation mediated by disease-associated microglia (DAM) and reactive astrocytes (Tuddenham et al., 2025; Zhang et al., 2026). In *C9orf72*-ALS, haploinsufficiency drives microglial vulnerability and dysregulates the broader neuroimmune response (Masrori et al., 2025). RIPK1-driven necroptotic pathways promote DAM formation, alongside a CNS-wide shift towards the MG2 microglial subtype, which is characterised by impaired oxidative phosphorylation (Tuddenham et al., 2025; Zelic et al., 2025). Concurrently, ALS astrocytes undergo disease-associated changes: they upregulate pro-inflammatory markers (*BHLHE40*, *EMP1*) whilst downregulating essential homeostatic genes required for neuronal health (*SLC1A2*, *SLC4A4*, *PTGDS*) (Pineda et al., 2024; Zelic et al., 2025). This central inflammation is exacerbated by peripheral immune dysregulation; *C9orf72*-ALS CD14 + monocytes upregulate interferon-stimulated genes (*IFITM2/3*), a complement-inflammation response that spatial transcriptomics maps directly onto areas of MN loss (Zhang et al., 2026).

Finally, sc/snRNA-seq reveals critical failures in oligodendrocytes, which provide essential metabolic support and myelin sheaths for axonal potential propagation (Narine and Colognato, 2022). During ALS progression, oligodendrocytes downregulate myelination and axonal guidance pathways, directly impairing the long-range projecting ET CSMNs that rely on them (Limone et al., 2024; Pineda et al., 2024). This is coupled with the suppression of metabolic and proteostasis factors, as well as a dysregulation of maturation-related genes (*SOX8*, *OLIG1/2*) (Pineda et al., 2024). Compounding these functional deficits, absolute oligodendrocyte numbers decline as the disease progresses, whilst neuroinflammatory myeloid cells increase (Zhang et al., 2026). This leaves an already damaged MN population without adequate structural or metabolic support, accelerating pathogenesis (Pineda et al., 2024; Zhang et al., 2026).

Collectively, these high-resolution studies map a complex convergence of intrinsic neuronal vulnerability, glia-mediated neuroinflammation and support-cell failure, as prime features in ALS. However, post-mortem patient tissue only offers a snapshot of the transcriptional states in surviving cells, limiting our understanding of spatiotemporal cellular dynamics that take place during ALS progression (Chang et al., 2025). On top of this, cryopreservation artefacts and molecular aberrations stemming from increased post-mortem intervals (PMI) prior to freezing further compound scRNA-seq analyses in post-mortem tissues (Giovannini et al., 2024).

In contrast to patient tissue, ALS model systems like mice, iPSCs or *Drosophila* facilitate tissue sampling across multiple timepoints, modelling cell subpopulation dynamics and gene expression across disease progression (Zhou et al., 2024). Even when tissue is frozen, this is done at the points of death and samples are prepared from larger populations, allowing for more accurate identification of true disease signal and minimising inter-individual differences. In this Review, we focus on how scRNA-seq/snRNA-seq within ALS model systems has supplemented findings from post-mortem data and identified novel mechanisms, helping drive our understanding of ALS pathophysiology.

## 2 Using scRNA-seq and snRNA-seq in ALS models

A number of recent studies have applied scRNA-seq techniques to ALS model systems, both *in vivo* and *in vitro* (Table 2). Here, we review these studies, focusing on how single-cell/single-nuclei transcriptomics is used in *Drosophila*, mouse and hiPSC ALS neuronal models to identify novel ALS neurodegenerative mechanisms (Liu et al., 2020; Ho et al., 2021; Schweingruber et al., 2025).

Mice and flies offer several key advantages for scRNA-seq analyses such as short generation times and brief lifespans, allowing scRNA-seq analysis at multiple timepoints, across the entire disease progression, helping to identify cell-type specific vulnerability and transcriptional dynamics (Layalle et al., 2021; Bonini, 2022; Nitta and Sugie, 2022; Shen et al., 2025). In *Drosophila* this is coupled with low costs of experiments and a vast genetic toolkit, allowing for inferences from the transcriptional analysis to be causally assessed (Shen et al., 2025). *Drosophila* models however lack the complex vertebrate-style glial immune interactions that modulate ALS pathogenesis, along with the long MN axons found in humans, limiting the translatability of findings related to non-cell-autonomous neuroinflammation or axonal transport stress (Table 4) (Hegde and Srivastava, 2022; Leggett et al., 2026).

**Table 4:** Summary of advantages and limitations of selected ALS model systems for scRNA-seq.

ALS model system	Advantages	Limitations
*Drosophila*	Facile genetics Huge availability of genetic tools Low costs, short generation time/lifespan Ability to sample across disease progression	Lack of complex mammalian immune system and neuronal inflammation Axons are not myelinated
Mouse	Mammalian nervous system Ability to look at role of non-neuronal and peripheral cells Ability to sample across disease progression	Cell specific differences in pathology compared to human (e.g., corticospinal neurons) High costs of running long term experiments Models often rely on over-expression and display very aggressive disease onset
hiPSC	Easy genetic modification Can model early disease state Can model both familial and sporadic ALS patient pathology Human model	Cell culture variability compounds batch effect correction Patient-specific genetic variation can distort scRNA-seq analyses Cannot model the effects of ageing

Mouse models, on the other hand, can help map non-neuronal contributions and glial dynamics within a complex and reproducible mammalian nervous system (Chen L. X. et al., 2024; Gautier et al., 2026). Nevertheless, differences in corticospinal tract structure mean that degenerating cortical single-cell profiles do not faithfully recapitulate human upper MN pathology, limiting scRNA-seq insights into MN degeneration (Fisher et al., 2023). Moreover, due to rapid disease progression early transcriptional kinetics may become distorted and obscured to scRNA-seq, limiting insight into these early cell-specific disease phases (Table 4) (Jagaraj et al., 2025).

Patient-derived hiPSC ALS models recapitulate human genomic landscape and can help model these very early disease states of both sALS and fALS pathologies, helping uncover early convergent gene dysregulations across multiple ALS pathologies but have a limited ability to reproduce ageing features, since they retain an embryonic or very young transcriptional state (Ardhanareeswaran et al., 2017; Dawoody Nejad and Pioro, 2025). Moreover, reproducibility issues between different cell culture batches and patient-specific genomic variations may increase variability and make batch-effect correction for analysis challenging (Table 4) (Dawoody Nejad and Pioro, 2025).

Combining the strengths of different model systems, whilst acknowledging their respective limitations, provides a powerful engine for discovery. This is especially true when integrated with patient data to prioritise disease-relevant pathways previously masked by bulk tissue analysis.

### 2.1 scRNA-seq and snRNA-seq analysis of ALS models

#### 2.1.1 ScRNA-seq in *Drosophila* models

scRNA seq analysis in *Drosophila* models has been extensively used to study developmental trajectories of cell populations during differentiation and their corresponding gene regulatory networks (Li, 2021). Similarly, single cell analysis has been used to explore brain ageing, revealing age-associated, cell-specific transcriptional changes in over 200 different neuronal and glial populations (Davie et al., 2018). In particular, mitochondrial turnover and respiration reduced with age, with a strong correlation between age and mitochondrial homeostasis in dopaminergic neurons and mushroom body Kenyon cells (KC) (Davie et al., 2018). Given that mitochondrial dysregulation has been implicated in ALS, it is possible that age-associated reductions in mitochondrial activity could precipitate ALS pathologies (Bradford and Rodgers, 2024).

These studies demonstrate the power of using scRNA-seq approaches to decipher complex cell specific gene expression signals across the *Drosophila* life course (Davie et al., 2018). Recently, this has been leveraged in a *C9orf72*-ALS *Drosophila* model, revealing novel pathological mechanisms of ALS (Table 1, Model No. 1) (Shen et al., 2025). Here, 10X genomics-based analyses on flies expressing 36 G_4_C_2_ repeats (C9) in neurons across multiple timepoints identified both vulnerable and resistant neuronal subpopulations, suggesting neurons display differential vulnerability to C9-repeat toxicity, highlighting the heterogeneity of cell-type specific responses to disease, something that would have been challenging with bulk RNA-seq alone (Molla Desta and Birhanu, 2025; Shen et al., 2025). The study also found that vulnerability was mostly determined by the basal transcriptional state of cells, rather than the response they mount, and that this was mostly associated with proteostasis genes (Shen et al., 2025). They went on to show that the Xbp1 transcription factor modulated the resistance to C9 toxicity, suggesting that ER proteostasis activity may be a key determinant of neuronal vulnerability in ALS (Shen et al., 2025). These findings correlate with those in patients brains showing that vulnerability of neurons mapped to transcriptional state, extending our previous understanding of the cell-specific mechanisms and, suggesting that proteostasis pathways may also play a role alongside mitochondrial and ribosomal dysregulation (Pineda et al., 2024).

#### 2.1.2 ScRNA-seq and snRNA-seq in mouse models

In mice, multiple studies leveraging the transgenic SOD1-G93A mouse model have converged on the critical role of neuroinflammation and microglia in disease progression (Table 1, Model No. 4–6).

Keren-Shaul et al. (2017) were the first to leverage scRNA-seq in this model, using massively parallel MARS-seq on spinal cord tissue during early (day 80) and late (day 135) disease stages. By relying on automated, unsupervised sampling rather than established markers, they identified a previously unknown subpopulation of DAMs from a heterogeneous cellular pool (Jaitin et al., 2014; Keren-Shaul et al., 2017). They found a 24% increase in DAM proportions in late-stage mice, suggesting these cells accumulate as the disease progresses. Crucially, this DAM accumulation was subsequently confirmed in ALS patient tissue, validating the use of mammalian mouse models for studying ALS neuroimmune pathologies (Tam et al., 2019).

Building on this, Chen L. X. et al. (2024) used snRNA-seq across multiple CNS regions (brain stem, spinal cord, cerebrum) to map the spatiotemporal trajectory of these DAMs (Chen L. X. et al., 2024). Their pseudo time analyses revealed that DAMs increase significantly during phases of accelerated MN death. By mapping this evolution, they identified that the downregulation of resting homeostatic markers, such as *Cx3cr1* and *P2ry12*, acts as a major turning point, leading to the downregulation of an inhibitory checkpoint, thus triggering DAM hyperactivation. This correlated with an upregulation of inflammation and endocytosis genes, leading to enhanced phagocytotic activity confirmed by *in vitro* assays. These functional shifts correlated with scRNA-seq datasets from ALS patients, which identified parallel pathogenic homeostatic genes’ downregulation (Tuddenham et al., 2025).

Subsequent spatial transcriptomic and longitudinal snRNA-seq data from SOD1-G93A mice brought these findings into a structural context (Table 1, Model No. 5). Gautier et al. (2026) demonstrated that microglial accumulation near MNs directly coincides with DAM gene upregulation and homeostasis gene downregulation. Furthermore, they found that the MNs themselves undergo distinct molecular changes, transitioning into a “disease-associated motor neuron” (DM) state characterised by downregulated axonogenesis, synaptic transmission and, ultimately, apoptosis (Gautier et al., 2026). Regulatory analyses identified CREB3 and ATF3 as key transcription factors driving this DM transition, suggesting that SOD1 mutations drive both DAM hyperactivation and cell-autonomous pathogenic transformations in MNs.

Beyond neuroinflammation, transcriptomic studies across different ALS models have highlighted neuronal hyperexcitability and ion channel dysfunction as shared pathogenic pathways. Using Drop-seq on the pontine brainstem of SOD1-G93A mice, Liu et al. (2020) identified distinct alterations in interneuron populations (INT1-3) and MNs (Table 1, Model No. 5) (Liu et al., 2020). Specifically, they found an upregulation of sodium channel genes (*Scn2a*) and a downregulation of potassium channel genes (*Kcna1* and *Kcnc3*) in MNs, driving hyperexcitability and calcium dysregulation that likely contribute to MN death. Interestingly, this hyperexcitability phenotype is mirrored in TDP43 loss-of-function models. Thapa et al. (2025) utilised snRNA-seq on the medial prefrontal cortex of *Tdp-43F/F* mice following selective TDP43 depletion (Thapa et al., 2025). They found that TDP43 loss induces severe GABAergic neuronal loss and calcium homeostasis dysregulation. Importantly, these model-based findings directly link to studies on human patient tissue, where TDP43 loss-of-function is associated with increased mis-splicing of the *KCNQ2* potassium channel, leading to intrinsic neuronal hyperexcitability (Joseph et al., 2025). Furthermore, GWAS in ALS patients have recently confirmed the widespread clinical relevance of these ion channel and vesicle-mediated transport pathways (Arreola-Aldape et al., 2025; Joseph et al., 2025).

Furthermore, TDP43 depletion models also showed an upregulation of DAM marker genes (*Oasl2* and *Lyz2*), linking TDP43 pathology back to the sustained DAM activation and gliosis observed in the SOD1 models (Chen L. X. et al., 2024; Thapa et al., 2025). Alongside SOD1 and TDP43, snRNA-seq has been applied to *C9orf72* models to uncover unique stress pathways. Analysis of the frontal cortex in *C9-BAC* and *C9orf72* knockout mice (Table 1, Model No. 2) revealed that progressive loss of *C9orf72* leads to the downregulation of ribosomal proteins and biogenesis machinery (Ma et al., 2023). This dysregulation induces a profound cellular stress response linked to proteotoxicity (Tye et al., 2019; Ma et al., 2023). Finally, Liu et al. (2020) additionally identified organophosphorus response pathways in Schwann and ependymal cells of SOD1-G93A mice, suggesting a link between genetic mutations and environmental insult vulnerability, though direct evidence across other models remains limited (Liu et al., 2020).

Overall, the pinpointing of cell-type-specific mechanisms, from DAM trajectories to hyperexcitability and ribosomal stress, highlights the power of single-cell approaches in disease discovery. However, despite the availability of FUS-ALS mouse models, current scRNA-seq analyses have been restricted almost entirely to SOD1-G93A, *Tdp-43F/F* and *C9orf72* models (Todd and Petrucelli, 2022). Applying these high-resolution techniques to FUS models is a critical next step to gain a truly holistic understanding of the mechanistic interplay between diverse ALS mutations.

#### 2.1.3 ScRNA-seq and snRNA-seq in hiPSC models

Ho et al., were the first to use scRNA-seq to thoroughly characterise an array of patient-derived *C9orf72-*HRE models, alongside sporadic and gene edited iPSC, to identify common ALS signatures and minimise the issue of heterogeneity of samples (Table 1, Model No. 3) (Ho et al., 2021). By sampling interneurons, cranial and spinal MN across timepoints during the *in vitro* differentiation they identified transcriptional changes linked to differentiation and cell-type specification, revealing early and predictive ALS expression signatures that could accurately distinguish between control and ALS hiPSC-MNs, validating them as potential predictive markers of disease-associated MNs (Ho et al., 2021). Interestingly, these signatures highlighted pathways overlapping with those in ALS post-mortem bulk RNA-seq data (Ho et al., 2021). For example the common downregulation of *ELAVL3, NUAK1*, *ADCYAP1*, *NDUFAF5*, *NNAT* and *DNMT3B*, genes linked to axon guidance and neurite growth, suggest that disruptions of homeostatic processes might occur during early MN development, priming these neurons for pathogenesis later in life (Ho et al., 2021). However, the droplet-based assays used in this study provided only around 100,000 reads per cell, limiting the number of DEG that can be accurately identified within each cell and therefore also the possibility of identifying novel ALS pathways (Ho et al., 2021; Namboori et al., 2021).

To overcome this limitation, subsequent ALS-hiPSC studies have utilised automated microfluidic technology (Fluidigm C1) to process up to 96 cells simultaneously, using long-distance PCR to full length mRNA transcripts, generating over 1.5 million reads per cell and sequencing each cell to a greater depth (Gong et al., 2018; Namboori et al., 2021). This increased depth allowed researchers to identify over three times as many genes than in normal droplet studies, helping dissect cell-type-specific TF networks in degenerating SOD1-E100G ALS patient-derived hiPSC-MNs in greater detail (Table 1, Model No. 6) (Namboori et al., 2021). Transcriptional regulatory network analyses on SOD1-E100G hiPSC-MN DEGs identified transforming growth factor β (TGF-β) signalling and downstream SMAD2 activation as key drivers of *SOD1*-E100G hiPSC-MN degeneration (Namboori et al., 2021). *In vitro* assays confirmed increased SMAD2 nuclear phosphorylation in both degenerating SOD1-E100G hiPSC-MNs and FUS-P525L hiPSC-MNs, with TGF-β inhibition enhancing cell survival, showing how key developmental pathways may be implicated in ALS MN gene dysregulation (Namboori et al., 2021).

Alongside hiPSC-MN cultures, researchers have also used snRNA-seq to show that ALS-linked *C9orf72* mutations severely impair microglial immune responsiveness, preventing their transition to a necessary reactive state which could contribute to disease pathology. Investigating patient-derived microglial xenografts in immunodeficient mice, snRNA-seq analyses revealed that *C9orf72*-HRE and knockout models downregulate immunoreactive HLA genes (*HLA-DPA1*, *XACT*) whilst upregulating homeostatic genes (*AFF3*, *DISP1*), findings cross-validated using *C9orf72*-ALS patient spinal cord and motor cortex tissues (Masrori et al., 2025). Corroborating this diminished *in vivo* reactivity, *in vitro* scRNA-seq of microglia/MN co-cultures stimulated with lipopolysaccharides (LPS) demonstrated an impaired cytokine response, reduced NF-κB signalling and the complete loss of an LPS-responsive microglial subpopulation in *C9orf72* mutant models (Gao et al., 2026). Together, these studies confirm that *C9orf72* haploinsufficiency prevents microglia from transitioning to a necessary reactive state. By failing to neuronal maintenance and actively contribute to ALS pathology (Masrori et al., 2025; Gao et al., 2026).

More recent scRNA-seq studies have also successfully paired ALS patient-derived neurons with isogenic, CRISPR/Cas9-edited hiPSC-MN lines harbouring ALS-linked FUS-P525L, FUS-R495X and *TARDBP*-M337V mutations to identify ALS mutation-specific pathways (Table 1, Model No. 9 and 12) (Schweingruber et al., 2025). *TARDBP*-M337V MNs showed a downregulation of non-coding RNA nuclear export whilst *FUS*-ALS mutations displayed downregulated genes involved in ER protein localisation (Schweingruber et al., 2025). Mitochondrial dysfunction, characterised by a broad downregulation of genes involved in ATP synthesis-coupled electron transport and oxidative phosphorylation, appeared as a common early pathological feature, prior to both TDP43 and FUS cytoplasmic mis-localization, suggesting mitochondrial impairments may drive aggregation in both *FUS*-ALS and *TARDBP*-ALS MNs (Schweingruber et al., 2025). Cross-comparisons with bulk RNA-seq data from *C9orf72*-ALS patient-derived MNs helped to identify shared mRNA metabolism and mitochondrial respiration dysregulation across *FUS*, *TARDBP* and *C9orf72-*ALS backgrounds (Schweingruber et al., 2025).

#### 2.1.4 Integrating scRNA-seq/snRNA-seq data from humans and model systems in ALS

Single-cell transcriptomics has profoundly advanced our understanding of ALS, yet neither human post-mortem tissue nor isolated model systems provide a complete picture on their own. Patient-derived tissue is unequivocally the most directly relevant source, capturing the true anatomical and genetic complexity of the human brain. However, it represents a static, end-stage snapshot of the disease that is often compounded by post-mortem artefacts (Giovannini et al., 2024). Conversely, model organisms (such as mice and *Drosophila*) and hiPSC cultures are living systems. Their distinct advantage lies in the ability to capture disease progression longitudinally across multiple timepoints, allowing researchers to track dynamic transcriptomic shifts from early presymptomatic stages down to late-stage neurodegeneration (Ho et al., 2021; Shen et al., 2025).

To bridge the gap between longitudinal disease mapping and true clinical relevance, researchers are increasingly cross-referencing living model data with patient datasets, integrating them using techniques such as the Expression Weighted Cell-type Enrichment (EWCE) or DEG comparisons (Liu et al., 2023; Shen et al., 2024). Such cross-species validations are critical, not only to provide novel mechanistic insights into ALS, but also cross-validate findings from multiple different sources (Liu et al., 2023; Shen et al., 2024).

Currently, this approach is most effective when comparing mouse and human datasets, given the conserved genomes, neural architecture and cellular pathways shared between mice and humans (Todd and Petrucelli, 2022). DEG cross-comparisons between human and *SOD1*-G93A mouse data identified several novel potential ALS biomarkers for non-neuronal microglia, including *CALM1*, *CALM2* and *HSPA1A*, which could aid effective ALS diagnosis and identification (Shen et al., 2024). These analyses also highlighted that microglia downregulate pathways related to thermogenesis and ribosomal function, reinforcing the link between energy metabolism and ALS pathogenesis (Shen et al., 2024). Furthermore, increased microglial oxidative phosphorylation correlated with a decline in intercellular interaction with astrocytes, driving microglial decline and worsening neuroimmune dysregulation. Given the role of toxic, cytokine-secreting M1 microglia in neuroinflammation and neuronal death, these cross-species findings strengthen the rationale for microglia-targeted therapies in ALS (Shen et al., 2024).

Similarly, EWCE comparisons across multiple large-scale mouse and human scRNA-seq datasets helped identify novel neuronal subpopulations involved in human ALS pathogenesis, such as spinal cord α- and γ-MN subpopulations: resistant γ-MNs survive in *SOD1*-G93A mice whilst vulnerable α-MNs degenerate (Lalancette-Hebert et al., 2016; Liu et al., 2023). The cross-comparisons also reveal γ-MNs associate primarily with genes known to carry ALS causal mutations (*SOD1*, *CHMP2B* and *VCP*), whilst α-MNs associate with susceptibility genes (Liu et al., 2023). Together, these integrative studies deepen our understanding of the regulatory mechanisms of ALS pathogenesis (Liu et al., 2023; Shen et al., 2024). Despite these advances, dataset integration between human and model systems in ALS research remains limited to mouse data, as the high degree of genetic and cell-type conservation between them, makes genetic and single cell-level cross-comparisons more straightforward (Breschi et al., 2017; Todd and Petrucelli, 2022). Current bioinformatic pipelines for aligning cell identity across divergent organisms are far from ideal. Reconciling highly conserved human cell types with non-mammalian counterparts, such as those in *Drosophila*, which lack exact anatomical equivalents, poses a formidable computational challenge. However, given the recent surge in *in vivo* fly scRNA-seq datasets, refining these alignment algorithms is essential. Future studies must develop better analytical pipelines to successfully map non-mammalian cell identities to human data, allowing the rapid, temporal discoveries made in simpler models to be accurately validated against true human pathology. Conversely, common mechanisms found in patient and mouse data, such as the differential roles of α- and γ-MNs in pathogenesis, could then be experimentally validated in more genetically tractable models (Liu et al., 2023; Shen et al., 2024).

In summary, cross-model single-cell transcriptomic comparisons reveal both convergent pathogenic axes and genotype- or model-specific mechanisms (Table 2). Conserved cross-model hallmarks include early mitochondrial and bioenergetic collapse, proteostatic vulnerability, involving modulated ER stress capacity and impaired ribosomal biogenesis, disrupted neuronal signalling characterised by ion channel dysregulation, altered intrinsic firing properties and defective calcium homeostasis (Liu et al., 2020; Ma et al., 2023; Schweingruber et al., 2025; Shen et al., 2025; Thapa et al., 2025). Glial reactivity is another recurring feature, though its phenotype diverges across genetic backgrounds: SOD1 and TDP43 models predominantly display hyperactive DAMs, whereas *C9orf72*-HRE models exhibit microglial immunoparalysis (Chen L. X. et al., 2024; Masrori et al., 2025). Conversely, other pathways diverge along distinct genetic lines, including aberrant TGF-β/SMAD2 signalling in SOD1-E100G hiPSC-MNs, impaired non-coding RNA nuclear export in *TARDBP*-M337V MNs and organophosphorus response pathways in SOD1 peripheral glia (Liu et al., 2020; Namboori et al., 2021; Schweingruber et al., 2025). Together, scRNA-seq cross-comparisons across multiple ALS model systems therefore can help identify both broad and genotype-specific disease mechanisms, giving crucial insight into the intrinsic heterogeneity of ALS (Scarcella et al., 2025).

## 3 Discussion and future directions

Single-cell and single-nucleus transcriptomics (scRNA-seq/snRNA-seq) have transformed our understanding of ALS by resolving cell-type-specific vulnerability and transcriptional dynamics (Pineda et al., 2024; Zelic et al., 2025). Whilst post-mortem tissue profiling has historically uncovered end-stage cellular attrition, it provides only a static cross-section of terminal pathology, obscuring the temporal sequences and early spatiotemporal dynamics that drive disease onset (Chang et al., 2025). Living model systems, spanning hiPSC-derived MNs, *Drosophila* and murine models, have partially bridged this temporal gap (Zhou et al., 2024; Dawoody Nejad and Pioro, 2025). Single-cell resolution in these systems has revealed dynamic temporal trajectories, identified vulnerable MN subpopulations, and cell-autonomous and non-cell-autonomous cascades (Liu et al., 2020; Ho et al., 2021; Liu et al., 2023; Chen L. X. et al., 2024; Schweingruber et al., 2025; Shen et al., 2025; Gautier et al., 2026). By providing high-resolution dissection of regulatory modifiers (such as TGF-β signalling and Xbp1 activity) and reactive glial substates, these platforms capture cell-type-specific phenomena that remain masked in bulk transcriptomic analyses (Namboori et al., 2021; Molla Desta and Birhanu, 2025; Shen et al., 2025).

Despite these insights, current single-cell investigations are fundamentally constrained by the limitations of the disease models themselves. Whilst hiPSC-derived MN and glia offer human genetic backgrounds and early developmental windows, cultures represent an artificial cellular environment, lacking the complex inter-organ interactions that shape organismal response to disease (Dawoody Nejad and Pioro, 2025). Moreover, they display high experimental variability due to donor heterogeneity as well as differing differentiation protocols, only partially helped by using isogenic CRISPR-edited lines, which may obscure relevant disease signatures (Hong et al., 2023). *In vivo* models, such as mice and flies, can help overcome these limitations, reproducing complex intra-tissue environment during disease progression, yet single-cell studies remain heavily skewed towards transgenic overexpression models, most prominently high-copy SOD1-G93A mice (Gurney et al., 1994; Zhou et al., 2024). Supra-physiological transgene expression can lead to high-copy artefacts, skewing resultant cellular phenotypes towards artefactual stress responses that do not accurately mirror endogenous disease initiation (Joyce et al., 2015). It will be important to expand single-cell profiling to endogenous knock-in lines and underrepresented fly and mice models (such as *TARDBP*, *FUS* and *C9orf72*) (Swarup et al., 2011; Qiu et al., 2014; Joyce et al., 2015; Langellotti et al., 2016).

In all cases, disease models cannot realistically replicate the cumulative lifetime environmental exposures (such as pesticides, organophosphates and heavy metals) implicated in sporadic ALS (Bozzoni et al., 2016). Consequently, single-cell responses to complex gene–environment interactions remain largely unmapped.

In any case, combining single-cell datasets across multiple model systems and human post-mortem cohorts provides an effective filter to eliminate model-specific artefacts and isolate high-confidence molecular signatures. When distinct genetic models (e.g., *FUS*, *TARDBP*, *SOD1*) converge on identical cell-type-specific stress states, such as early mitochondrial respiratory decline or specific reactive microglial states, these shared signatures represent prime targets for translational discovery although single cell analysis remains correlational and causal links need to be validated across disease models (Ho et al., 2021; Shen et al., 2024; Schweingruber et al., 2025).

A current challenge in treatment administration is the lack of reliable, sensitive genetic biomarkers for different ALS disease stages and pathophysiology (Roy et al., 2025). Single cell studies can dissect cell-specific ALS-associated molecular and transcriptomic signatures, which could then serve as potential disease biomarkers (Molla Desta and Birhanu, 2025). By isolating signatures specific to vulnerable MN or reactive neuroinflammatory states (e.g., unique DAM or reactive astrocyte clusters), researchers can identify candidate biomarkers for translation into accessible biofluids like cerebrospinal fluid (CSF) or plasma (Keren-Shaul et al., 2017; Liu et al., 2020; Namboori et al., 2021; Gautier et al., 2026). For example, cross-species profiling highlighting microglia ribosomal down-regulation and altered oxidative phosphorylation profiles in vulnerable MN could provide concrete targets to monitor disease status in patient biofluids (Shen et al., 2024).

However, screening for universally shared pathways risks erasing patient- and subtype-specific molecular signatures. ALS is clinically and pathologically heterogeneous; forcing diverse datasets into a single convergent consensus may obscure distinct, individualised pathogenic drivers that dictate clinical progression rates and therapeutic response (Scarcella et al., 2025). Rather than treating ALS as a monolithic clinical entity, single-cell signatures from diverse genetic models can be used to stratify patients by molecular signatures, as has been done for AD (Luquez et al., 2026). This analysis could identify clinically relevant disease-associated molecular signatures shared across different pathologies, such as mitochondrial/oxidative phosphorylation collapse or ribosomal homeostasis (Ho et al., 2021; Schweingruber et al., 2025). This stratification could facilitate targeted clinical trial enrolment, categorising participants based on active underlying biological pathways, such as primary neuroinflammation versus bioenergetic or proteostatic collapse, rather than broad clinical phenotypes alone (M. Amaral et al., 2024).

In addition to the limitations of ALS model systems, several technical challenges with current scRNA-seq and snRNA-seq approaches remain. ScRNA-seq cell extraction techniques can damage fragile or dying neuronal populations, leading to a disproportionate cell-type bias towards more resistant glial populations, skewing results and underrepresenting the very degenerating neurons of interest (Bakken et al., 2018). SnRNA-seq helps overcome these challenges to some extent by sampling more robust nuclei, but in doing so excludes cytoplasmic mRNA, limiting the detection of cytoplasmic processes such as mRNA metabolism, which play an important role in ALS development (Jovic et al., 2022). Furthermore, standard droplet-based platforms face a continuous trade-off between sequencing depth and cell throughput, frequently failing to capture low-abundance transcription factors or splice variants (such as CE inclusion) (Li, 2021). To address these gaps, scRNA-seq/snRNA-seq approaches could be combined with complementary modalities, such as spatial transcriptomics to help capture fragile cells *in situ*, ATAC-seq to identify cell-type-specific master regulatory switches, as well as single-cell and spatial proteomics to identify functional protein expression, capturing post-translational modifications, pathological protein mis-localization (e.g., nuclear TDP-43 clearance) and proteostatic failure (Guise et al., 2024; Scarcella et al., 2025; Gautier et al., 2026; Soltys et al., 2026).

In summary, integrating scRNA-seq/snRNA-seq with ALS model systems provides invaluable insights into ALS pathophysiology, revealing novel ALS-associated cell subtypes, cellular disease signatures and MN vulnerability modifiers. However, several challenges remain, including limitations with ALS models, intrinsic limitations of scRNA-seq/snRNA-seq techniques themselves and limited translational efforts so far (Bakken et al., 2018; Jovic et al., 2022; Zhou et al., 2024). To tackle these challenges, research efforts should firstly, move towards more integrated meta-analyses combining animals, cells and human cohorts, implementing computational frameworks that map functional analogues and homologues across species to separate core, conserved driver networks from model-specific artefacts. To do this successfully, scRNA-seq analytical pipelines should be standardised with robust preprocessing steps, allowing datasets from different model systems and patients to be compared to each other (Nesari et al., 2026). These meta-analyses should be coupled with multiple clinical cross-comparisons to help better link distinct and common cellular states to clinical phenotypes, something that would also be useful for the development of reliable cellular biomarkers (Roy et al., 2025). Finally, scRNA-/snRNA-seq should be further integrated with other omic approaches, such as spatial transcriptomics, proteomics and metabolomics, with integrated multi-omic datasets potentially facilitating a more holistic understanding of ALS pathophysiology from multiple angles (Krokidis, 2020; Guise et al., 2024; Scarcella et al., 2025; Gautier et al., 2026; Soltys et al., 2026).
